# Preparation and Evaluation of Exhaust-Purifying Cement Concrete Employing Titanium Dioxide

**DOI:** 10.3390/ma12132182

**Published:** 2019-07-07

**Authors:** Rui He, Xin Huang, Jiansong Zhang, Yao Geng, Haidong Guo

**Affiliations:** 1 School of Materials Science and Engineering, Chang’an University, Xi’an 710061, China; 2 School of Construction Management Technology, Purdue University, West Lafayette, IN 47907, USA; 3 Qinghai Academy of Transportation Sciences, Xining 810008, China

**Keywords:** photocatalytic pavement, vehicle exhaust, photocatalytic concrete, porous concrete, degradation efficiency

## Abstract

To address the increasing air pollution caused by vehicle exhaust, environment-friendly pavement materials that possesses exhaust-purifying properties were prepared using common cement concrete and porous cement concrete as the base of photocatalyst nano-titanium dioxide (TiO_2_), respectively. Firstly, Fe^3+^-doped TiO_2_ powder was prepared by applying planetary high-energy ball milling in order to improve the efficiency of the semiconductor photocatalyst for degrading vehicle exhausts. Two nano-TiO_2_, namely the original and modified nanomaterials, were adopted to produce the photocatalytic cement concretes subsequently. The physicochemical properties of the modified powder, as well as the mechanical and photocatalytic properties of TiO_2_-modified concrete, were characterized using a suite of complementary techniques, including X-ray diffraction (XRD), scanning electron microscopy (SEM), energy dispersive spectroscopy (EDS), compressive strength and degradation efficiency tests. The results show that the ball milling method not only successfully doped Fe^3+^ into catalysts but also caused significant changes in: (1) decreased particle sizes, (2) more amorphous morphology, (3) decreased percentage of the most thermodynamically stable crystal facet, and (4) increased percentage of other high gas sensing crystal facets. Both the original and modified nano-TiO_2_ can improve the concrete strength while the strengthening effect of modified nanomaterials is superior. It is pronounced that the photocatalytic property of the modified nano-TiO_2_ is much better than that of the original nano particles, and the degradation rate of porous concrete is also better than common concrete when exposed to the same photocatalyst content. In a comprehensive consideration of both mechanical performance and degradation efficiency, the recommended optimum dosage of TiO_2_ is 3% to 4% for exhaust-purifying concrete.

## 1. Introduction

With the acceleration of the urbanization process and car ownership, vehicle exhaust has become one of the main causes of air pollutions all over the world, especially in the developing and densely populated countries, such as China, for which the vehicle exhaust that comes from fossil fuels has accounted for about 25% of the total industrial exhaust emissions [1,2]. Air pollutants, mainly represented by nitrogen oxides (NO_x_) and sulfur oxides (SO_x_) in the emissions, can cause photochemical smog, haze, and acid rains. This poses great threat to ecosystems as well as the wellbeing of human and other species if it reaches a certain level of concentration in the atmosphere [3,4,5]. During the past decade, various countermeasures have been adopted to reduce vehicle emissions, such as using cleaner fuel, installing a gas-cleaning device in vehicles, and even developing electromobiles [2]. Nevertheless, vehicle exhaust is still a major problem, especially due to the rapid growth of the transportation sector.

As the nearest contact point after exhaust emissions, the vicinity of the road is where NO_x_ is prone to accumulate due to the gas flows through the exhaust devices and its higher density than oxygen. In street canyons or urban areas, the NO_x_ concentrations can be considerably higher [6,7]. Faced with this dramatic issue, innovative exhaust-purifying pavements, also known as photocatalytic or functional pavements, have been proposed to degrade NO_x_ and improve the air quality of road environment, by leveraging heterogeneous photocatalytic oxidations. As of now, photocatalytic pavements have become the most promising strategy to solve the road traffic pollutions, as confirmed by the large number of laboratory-scale tests and an increased amount of real scale studies over the past ten years [8,9].

Recently, photocatalytic oxidation was ranked among the most popular green chemical methods for sustainable development [10,11]. Amongst all the photocatalytic oxidations for NO_x_ degradation in photocatalytic pavements, nano-titanium dioxide (TiO_2_), most often anatase TiO_2_, is the most frequently used catalyst due to its low price, robustness, nontoxicity, and mild reaction conditions [9]. When activated by ultraviolet (UV) light and water, nano-TiO_2_ can convert the NO_x_ into harmless nitrates. It can degrade almost all organic matters in the vehicle emissions [3,8].

However, the anatase-type TiO_2_, which is proved to have strong photocatalytic degradation ability, is only active under high energy UV irradiation due to the large band gap of 3.2 eV, which composes less than 5% of the solar spectrum [8,12,13]. It means that the efficiency of this photocatalytic process for outdoor photocatalytic pavement is very slow. To address this issue, different methods have been attempted to improve the degradation efficiency, such as changing the TiO_2_ concentration or type, increasing the contact area between TiO_2_ and sunlight, and so on [6,8,14]. In spite of such exploration, the low efficiency still makes photocatalytic pavements difficult to be promoted.

In the research field of semiconductor materials, numerous studies have been undertaken to induce visible light activity into normally purely UV-active TiO_2_ by hydrothermal, sol-gel, the precipitation method, and other chemical and physical processes [13,15]. Doping with transition metal ions such as V, Cr, Fe, Co, and Ni by high voltage acceleration in the range of 50–200 keV could enable a large shift in the absorption band of these photocatalysts toward the visible light range, with differing levels of effectiveness [16]. This principle is expected to have a more promising outlook in improving the photocatalytic performances of TiO_2_. Thus, a large number of processing methods have been proposed for the modification of TiO_2_ according to the literature [17,18]. Among state-of-the-art techniques, the ball milling method makes mass production of catalysts more viable over other conventional chemical methods. This method has the advantages of low cost, environmental friendliness, and high efficiency and controllability in the laboratory, even in industry [16,19].

Porous concrete has a higher porosity than common concrete and is more susceptible to sunlight, thereby accelerating the photocatalytic reaction. In view of this situation, common cement concrete and porous cement concrete were both used to be the base of nano-TiO_2_ to prepare the green pavement material with photocatalytic function. In this paper, the commercial nano-TiO_2_ was first modified using the planetary ball milling technique to produce an Fe^3+^-doped TiO_2_ photocatalyst. Following that, two nano-TiO_2_, namely the original and modified nanomaterials, were adopted to produce the TiO_2_-modified cement concretes. The mechanical and photocatalytic properties of TiO_2_-modified concrete were studied. Promising results were obtained showing enhanced photocatalytic efficiency and compressive strength in the use of modified TiO_2_ comparing to original TiO_2_.

## 2. Experiments 

### 2.1. Materials

In this study, an anatase-type commercial nano-sized TiO_2_, labelled as original TiO_2_, was provided by Jiangsu Hehai Nanometer Science & Technology Co. Ltd (Taizhou, China). The detailed physical characteristics of this TiO_2_ are shown in Table 1. The modifier used for the Fe^3+^-doped TiO_2_ was analytically pure ferric nitrate (Fe(NO_3_)_3_⋅9H_2_O).

The Chinese 42.5 ordinary Portland cement from Jidong (Tangshan, China) was used to prepare concrete samples, along with coarse aggregate (maximum size of 20.0 mm) and fine aggregate (clean, natural sand, maximum size of 4.75 mm, fineness modulus of 2.3). The chemical agent superplasticizer from Subote (Nanjing, China), with a 25% solid concentration, was used at a dosage of 0.8% by mass of cement.

The grading of porous concrete coarse aggregate is the key factor affecting its permeability and mechanical properties. After determining the target porosity and water–cement ratio of concrete, the mixing ratio was calculated according to the volume method. The coarse aggregate gradation used in this study, combined with those in previous researches, is shown in Table 2 [20,21,22].

### 2.2. Methods

#### 2.2.1. Preparation of Modified TiO_2_

In contrast to the commonly used chemical modification method for nano-TiO_2_ that requires complex raw materials and operation processes, the planetary high energy ball milling method was used in this study to prepare the iron-doped nano-TiO_2_ photocatalyst, of which the process is simpler and the production is continuous. During the milling process, TiO_2_ powder undergoes severe plastic deformation due to strong collision, resulting in serious distortion of the lattice of TiO_2_, a large increase of internal defects, and improved catalyst activity. At the same time the ball milling process will produce a lot of heat, coupled with mechanical force to induce the separation of Fe^3+^ from ferric nitrate and its diffuse into the distorted TiO_2_ lattice, resulting in the formation of a new iron-doped photocatalyst which can reduce the probability of electron recombination and finally achieve the improvement of the photocatalytic performance [19,23].

**Figure 1:**
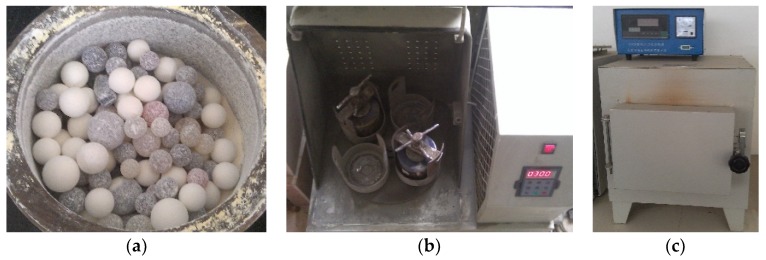
Planetary high energy ball milling setup. (**a**) Ball mill jar, (**b**) Planetary high energy ball mill, and (**c**) Muffle furnace.

**Figure 2:**
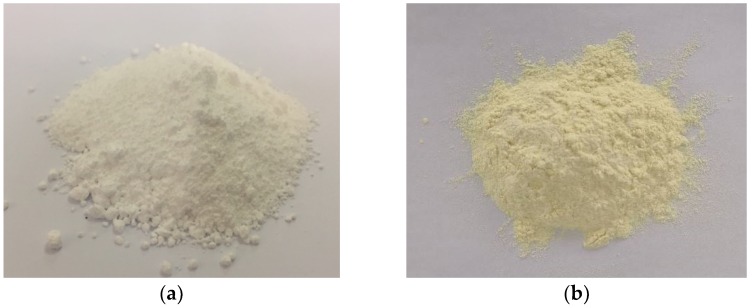
The original nano-TiO_2_ and modified nano-TiO_2_. (**a**) Original TiO_2_ and (**b**) Modified TiO_2_.

Based on the above principle, the preparation process involves firstly weighing nano-TiO_2_ and ferric nitrate in the amount of 100 g and 15 g, respectively. Then the starting materials were manually premixed and poured into the ball mill jar. The rotational speed and the ball-to-powder weight ratio were 300 rpm and 30:1, respectively. After 3 h of ball milling process, the mixtures were put into a muffle furnace, and calcined at 400 °C for 3 h. Subsequently, the obtained samples were washed by deionized water three times to remove any residual ferric nitrate. At last, the samples were dried at 90 °C for 6 h and then ground into powders. The high energy ball milling setup is shown in Figure 1. The original and modified nano-TiO_2_ are shown in Figure 2.

#### 2.2.2. Preparation of Concrete Samples

Two types of photocatalytic cement concretes were prepared, namely, the common concrete and the porous concrete, and both of the two TiO_2_ products (original and modified) were used for each type of concrete. That is to say, a total of four sets of photocatalytic concretes were prepared. Nanomaterials were used to replace the quantity of cement in the amount of 0%, 1%, 2%, 3%, 4%, and 5%, respectively. The common concrete reference ratio is cement:water:sand:gravel:water-reducing agent, 395:178:621:1205:4. The porous concrete reference ratio is cement:water:gravel:water-reducing agent, 252:100:1542:2. The experiment program is summarized in Table 3, and the contents for the two TiO_2_ products were the same.

The preparation process of concrete in this study is described as follows: Firstly, mixing the powder and coarse aggregate for 1 min in dry situation, then adding water slowly and continually mixing for 2 min, and finally forming a 100 mm × 100 mm × 100 mm cube test block. After 24 h of curing, cube test blocks were removed from molds and put into a standard curing room to keep curing at 20 ± 2 °C and relative humidity ≥ 95%. When the curing age arrived at 28 days, the blocks were taken out to carry on experiments of mechanical and photocatalytic properties. The compressive strength of each group of concrete samples was tested in accordance with T 0553 in JTG E30-2005 “Test Methods of Cement and Concrete for Highway Engineering” [24].

#### 2.2.3. Test of Photocatalytic Degradation Efficiency

**Figure 3:**
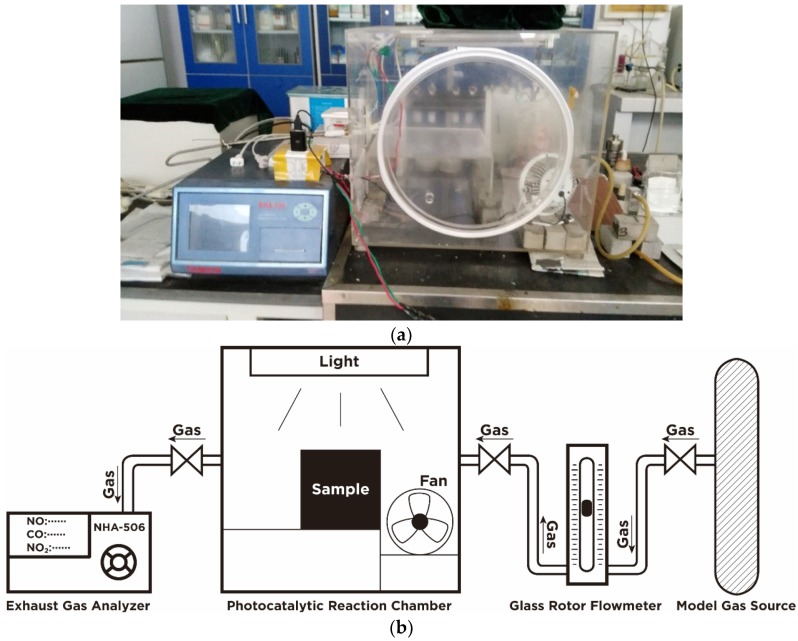
Photocatalytic degradation test equipment [26]. (**a**) Device appearance and (**b**) Device principle.

The photocatalytic degradation efficiency was tested in a custom-designed testing system that contains a continuous gas flow reactor according to the Italian Standard UNI 11247 [7,25], as shown in Figure 3 [26]. The test samples were the pre-prepared concrete blocks. The device mainly consisted of the following four parts: A sample reaction chamber, a simulation photo source, a standard gas source, and a gas analyzer. Among these four parts, the reaction chamber was made of borosilicate glass in order to prevent the chamber from absorbing pollutant gas and UV light. The surface of the samples placed in the reaction chamber was kept parallel to the lamp to ensure that the surfaces of samples were uniformly illuminated. The simulation photo source comprised of a mercury vapor lamp and an incandescent lamp on the upper side of the reaction chamber, with the wavelength range of 300 to 700 nm, which covers most of the visible range of sunlight [27]. The radiant energy was 48 W/m^2^, which is close to the sun radiation intensity at mid-latitudes [3]. On both side of the reaction chamber, there was a gas inlet and a gas outlet, respectively, which were used to supply gas for tests and extract gas for the analyzer. The gas analyzer was located at the same side with gas outlet. A small fan was placed at the corner of the reaction chamber to ensure test gas reacts completely on the specimen surface.

According to the main components of automobile exhaust gas, NO was used as the main pollutant model of photocatalytic degradation experiment. The concentration of NO used during the test was 200.0 ppb according to former studies [4,9]. The gas analyzer NHA-508 (Anhua, Foshan, China) was used as the detection device for NO concentration.

#### 2.2.4. Microstructure Analysis

The morphologies of the powdered TiO_2_ and the inner structure of concrete samples were observed with scanning electron microscopy (SEM, Hitachi S-4800, Tokyo, Japan). The surface elemental analysis of the samples was performed using energy dispersive X-ray spectroscopy (EDS) [13,28]. After the mechanical test, the samples subject to SEM analysis were taken from the fragments caused by the mechanical test, and conductive coating with gold was applied after drying treatment to prevent charging effects. In addition, the powder X-ray diffraction method (XRD, Bruker D8 Advance, Berlin, Germany) was employed to investigate the influence of modification on the crystal phase of the powdered TiO_2_ with Ni-filtered Cu Kα radiation from 15° to 80°.

## 3. Results and Discussion

### 3.1. Characterization of TiO_2_ Samples

**Figure 4:**
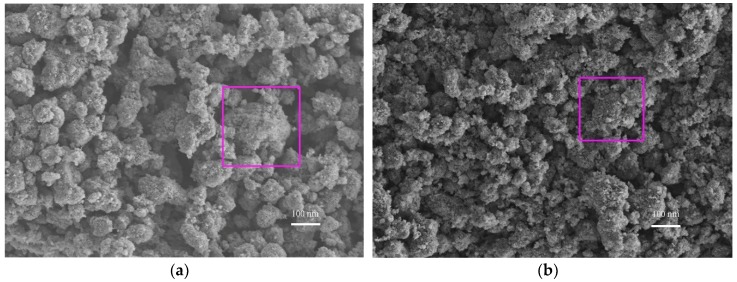
Microtopography of nano-TiO_2_. (**a**) Original TiO_2_ and (**b**) Modified TiO_2_.

**Figure 5:**
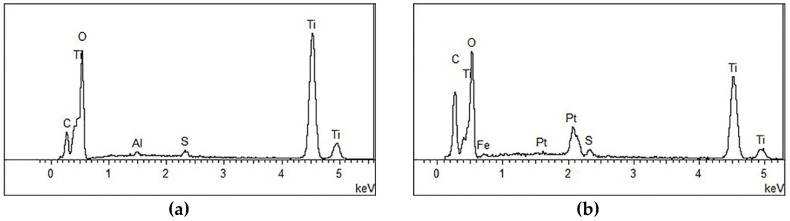
Energy Spectrum Analysis of nano-TiO_2_. (**a**) Original TiO_2_ and (**b**) Modified TiO_2_.

The effect of the planetary high energy ball milling treatment on the catalyst morphology and crystal structure was investigated by SEM and XRD tests, prior to preparing concrete samples. The SEM results are shown in Figure 4 and its energy spectrum is shown in Figure 5. It can be seen from Figure 4 that the amorphous nanoparticles were distributed unevenly and there were obvious agglomerations in both samples. However, the TiO_2_ particles were more prone to agglomeration after ball milling, which can be partially attributed to the reduction of particle sizes, as well as the high surface energy that was caused by ball milling. Therefore, the modified TiO_2_ appears to be more amorphous and finer, providing more surface area for the activation of photocatalytic activity. The EDS spectrum verifies that the ball milling procedure successfully introduces iron into TiO_2_ while the spectrum of original TiO_2_ has no iron.

**Figure 6:**
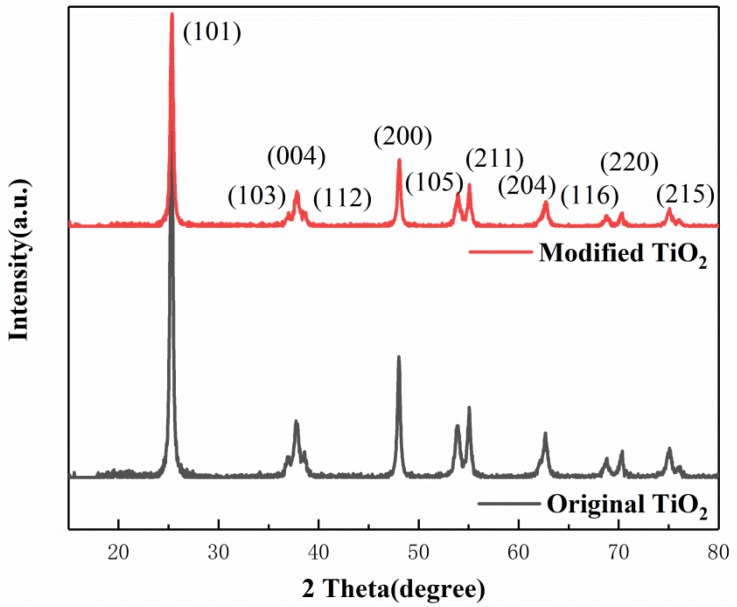
X-ray diffraction pattern of nano-TiO_2_.

According to the XRD patterns of the original and modified TiO_2_ shown in Figure 6, the diffraction peaks of both samples showed fingerprint features of an anatase TiO_2_ structure with no other characteristic peaks observed, indicating the phase purity of the samples. The percentage of diffraction intensity at different crystal facets is depicted in Table 4. It can be seen that the percentage of the (101) peak, which is the most thermodynamically stable and has relatively low chemical activity [19], decreases sharply from 51.84% to 48.47% after applying the ball milling procedure. The percentages of the other peaks for Fe^3+^-doped TiO_2_, such as (004), (105), (211), and (116), which have high gas sensing activity [29,30], are all increased to some degree. Correspondingly, the percentage of (101) crystal facet is decreased, which indicates that the preferred orientation of (101) has been compromised.

On the basis of these data, the average crystal sizes were calculated according to Scherrer’s equation [31]. For the original and modified TiO_2_, the results were 21.16 nm and 20.78 nm, respectively. It demonstrates that ball milling favors the formation of small crystallites, leading to more intense diffraction peaks, with the decrease of (101) crystal facet. In summary, it can be concluded that the planetary high energy ball mill can dope Fe^3+^ ions into nano-TiO_2_, making it successfully modified.

### 3.2. Mechanical Property Analysis

**Figure 7:**
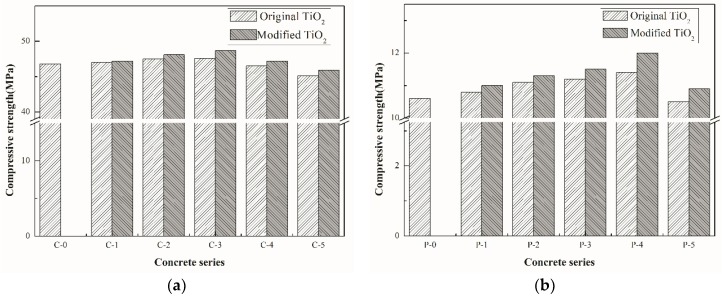
Results of 28 days compressive strength for each mix, (**a**) common concrete and (**b**) porous concrete.

The compressive strength test was conducted to ensure that the incorporation of nano-TiO_2_ did not compromise the mechanical performance of concrete, as shown in Figure 7. It can be seen from the figure that the compressive strength of two types of concrete both increased firstly and then reduced with the continuous increment of nano-TiO_2_ content. It indicates that the incorporation of nano-TiO_2_ is beneficial to the improvement of concrete strength, which is mainly due to the micro-filling effect of nano-TiO_2_, making the internal structure of concrete more compact and leading to the improvement of strength. The maximum increase in strength is about 3% and 5%, respectively. As the particle size of modified TiO_2_ is smaller than that of the original TiO_2_, the strength of modified TiO_2_ concrete is higher than that of the original TiO_2_ concrete with the same content of TiO_2_. In addition, the incorporation of Fe^3+^ can motivate the formation of iron-containing hydration products [32,33], which makes the internal structure more compact, and therefore improves the strength.

Nonetheless, when the nano-material content exceeds a certain range, the strength of each group begins to decline. At first, it can be ascribed to the agglomeration tendency as illustrated in Section 3.1, which could easily cause uneven distribution within the structure. On the other hand, the replacement of cement by TiO_2_ makes the corresponding cement content decrease when the nano-materials content increases excessively. In terms of the material strength, the best nano-TiO_2_ content of cement were 3% and 4% for the two types of concrete, respectively.

### 3.3. Photocatalytic Property Analysis

The photocatalytic degradation test of concrete was carried out using the custom-designed test device proposed in this study. It should be noted that the device was subjected to a blank control verification prior to the start of the formal test, and the change of NO concentration was recorded. During the equipment verification, NO concentration was almost unchanged, proving that the sealing of the reaction chamber was good. The parameters used in the testing process are shown in Table 5. Keeping the above test parameters remaining unchanged, photocatalytic degradation tests of all samples were conducted.

**Figure 8:**
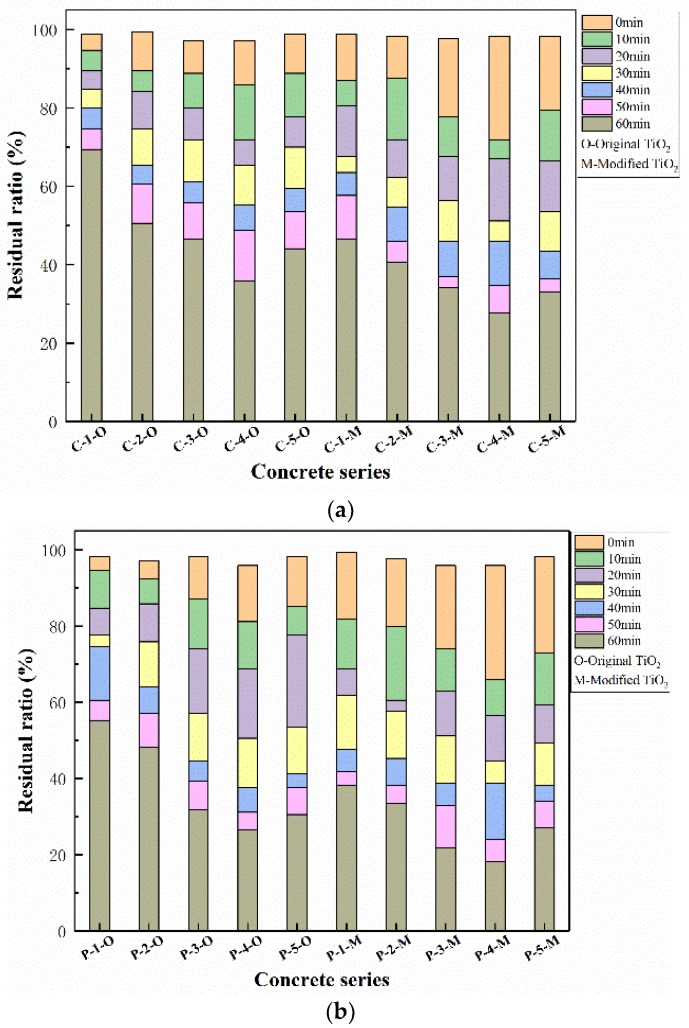
Photocatalytic degradation results of concrete in each group. (**a**) Common concrete and (**b**) porous concrete.

The residual rate of NO concentration after different periods of photocatalysis was calculated and displayed in Figure 8. It should be pointed out that the NO concentration results of concrete samples without TiO_2_ almost stayed constant. Thus, these results were not depicted in Figure 8. As it shows, the degradation rate of all the samples increases with the extension of irradiation time. Overall, the degradation rate of samples with modified nano-TiO_2_ was much higher than that of the original nano-TiO_2_ samples. The reason can be partially attributed to the smaller particle size that causes more contact area with pollutants. Also, the doped Fe^3+^ in the crystal of TiO_2_, by the mechanical chemistry theory of ball milling as mentioned above, can act as an acceptor which can trap photogenerated charges and reduce the metal ion species into a lower oxidation state. Then, due to the close energy level of the Ti^4+^/Ti^3+^ couple, the transformation of Ti^4+^ into Ti^3+^ is accelerated, which has been reported to improve the photodegradation by acting as a hole trap [15,34]. Another point is that Fe^3+^ dopant, which has been reported to be effective to the batho-chromic shift, promotes a possible extension of the activity of TiO_2_ towards visible light range [35,36]. Therefore, the photocatalytic activities of TiO_2_ are enhanced significantly.

**Figure 9:**
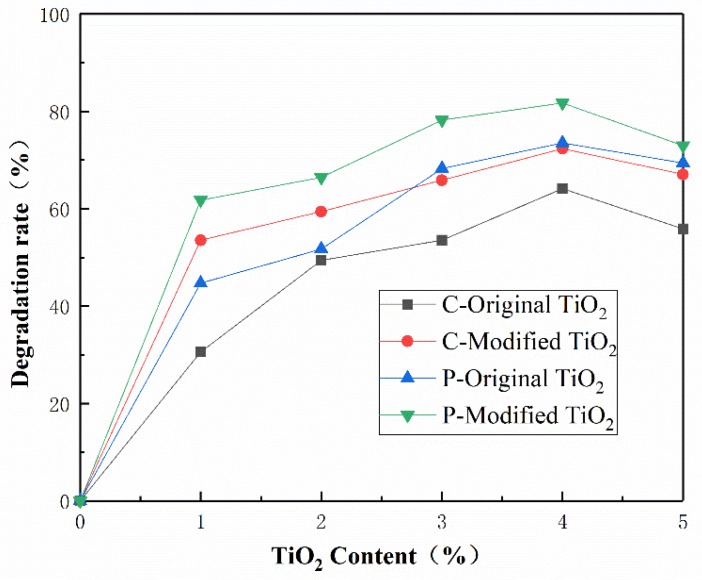
Comparison of photocatalytic degradation rate test results.

For further comparison, the photocatalytic degradation rates of all the samples at 60 min are shown in Figure 9. At the same content of nano materials, the degradation rate of concretes with modified nano-TiO_2_ is about 13% to 20% higher than that with original nano-TiO_2_. At the same time, the photocatalytic degradation rate of porous cement concrete is better than that of common cement concrete, which is because of the greater contact area with light caused by the connected pores of porous concrete. In the case of photocatalytic property, the optimized content of TiO_2_ is suggested to be 4%.

### 3.4. Microstructure Analysis of Modified Concrete

**Figure 10:**
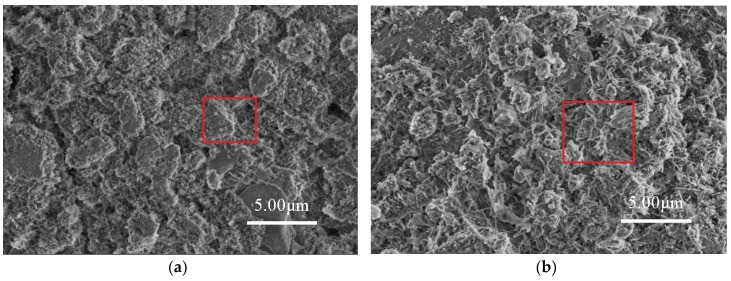
Microstructure analysis of concrete specimens. (**a**) C-0 and (**b**) C-4 with modified TiO_2_.

**Figure 11:**
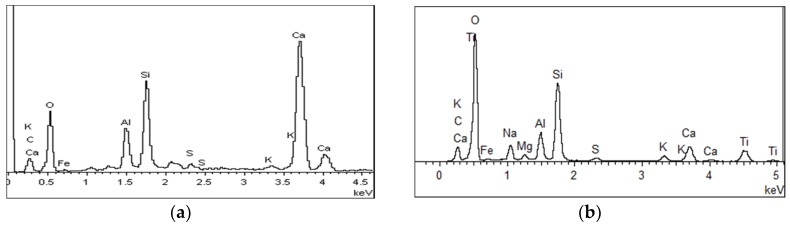
Energy spectrum analysis of concrete specimen. (**a**) C-0 and (**b**) C-4 with modified TiO_2_.

The microstructural test of C-0 and C-4 are shown in Figure 10. It can be seen from Figure 10 that the internal structure of C-0 concrete is looser, and the cracks or pores can be clearly seen between the hydration products. C-4 concrete is relatively dense inside, and agglomerated particulate matters are filled between the hydration products. In order to further confirm the composition of the material, the two samples were analyzed by EDS. The results are shown in Figure 11. It can be seen from Figure 11 that the main elements in C-0 are calcium, silicon, and oxygen, whereas for C-4, calcium, silicon, and oxygen elements, as well as a lot of titanium. It indicates that the inner structure of C-4 has titanium-containing substances except common hydration products, which is TiO_2_. Therefore, based on the above analysis, it can be seen that the introduction of modified nano-TiO_2_ can be filled in the cracks or pores between the hydration, so that the structural density increased, which in turn improves the strength of C-4.

In this paper, we can see that the introduction of nano-TiO_2_, especially the modified nano-TiO_2_, has a positive impact on the mechanics and degradation properties of concrete. But there is an optimal amount. Considering the effects of mechanical properties and degradation efficiency, the recommended optimum dosage is 3% to 4%.

## 4. Conclusions

This paper highlights the feasibility of using the planetary high energy ball milling method in modifying TiO_2_, as well as subsequent preparation of exhaust-purifying cement concrete. The photocatalytic efficiency and mechanical performance of exhaust-purifying cement concrete were both evaluated. The main conclusions can be summarized as follows:

(1) The modified nano-TiO_2_ doped with Fe^3+^ was successfully prepared by planetary high energy ball milling. The ball milling process caused significant changes to the feature parameters of TiO_2_, including decreased particle size, the more amorphous morphology, the compromised percentage of the most thermodynamically stable crystal facet, and the increased percentage of other high gas sensing crystal facets.
(2) The incorporation of nano-TiO_2_ is beneficial to the improvement of concrete strength because of the micro-filling effect of nano particles. The incorporation of Fe^3+^ can motivate the formation of iron-containing hydration products, leading to the further increase of concrete strength. In terms of the material strength, the best nano-TiO_2_ content of cement was 3% and 4% for the two types of concrete, respectively.
(3) The employment of nano-TiO_2_ endow the concrete the functional properties of purifying exhaust gas. Due to the doping of Fe^3+^ in the crystal, the photocatalytic degradation effect of modified nano-TiO_2_ is better than that of the original nano-TiO_2_. The degradation rate of porous concrete is much higher than that of common concrete with the same content of photocatalyst. For both types of concrete, the photocatalytic degradation rate comes to the peak when the nano-TiO_2_ content is 4%.
(4) The internal structure of the concrete was modified to be more compact with the adoption of nano-TiO_2_, so the strength is improved. In a comprehensive consideration of both mechanical performance and degradation efficiency, the recommended optimum dosage of TiO_2_ is 3% to 4% for concrete. It is the most promising strategy to develop functional pavements using photocatalytic porous concrete to purify the urban traffic pollutions.

At last, it is strongly suggested that the modified nano-TiO_2_ doped with Fe^3+^ may be further fused with other granular metamaterial components in concrete, to explore potential enhancement in both mechanical and chemical properties in further studies on the basis of the dispersive behavior of granular materials [37,38].

## Figures and Tables

**Table 1:** Technical index of nano-TiO_2_.

Appearance	Average Particle Size/nm	Specific Surface Area/m^2^⋅g^−1^	Crystal Type	TiO_2_ Content/%	Photocatalytic Efficiency/%	pH Value
White powder	10	70.2	Mixed crystal type (anatase, rutile)	>92	≥68	Partial acidity

**Table 2:** Coarse aggregate gradation of porous concrete.

Sieve/mm	2.36	4.75	9.5	13
Passing Rate/%	0	6.5	85.7	100

**Table 3:** Experiment program.

Concrete Type	Common Concrete						Porous Concrete					
Nano-TiO_2_ content/%	0	1	2	3	4	5	0	1	2	3	4	5
Code	C-0	C-1	C-2	C-3	C-4	C-5	P-0	P-1	P-2	P-3	P-4	P-5

**Table 4:** The percentage of diffraction intensity at different crystal facets.

Crystal Facets	Percentage (%)								
	(101)	(103)	(004)	(112)	(200)	(105)	(211)	(204)	(116)
Original TiO_2_	51.84	1.35	5.75	1.45	16.23	6.48	9.02	5.75	2.13
Modified TiO_2_	48.47	2.57	7.51	2.52	15.03	7.03	9.21	5.48	2.18

**Table 5:** Photocatalytic degradation test parameters.

Total Amount of Pollutant Gas	Flow Rate	Relative Humidity	Detection Time	Concrete Age
12 L	1.2 L/min	50%	10 min/once total 1 h	28 days
